# Velvet Antler Mobilizes Endothelial Progenitor Cells to Promote Angiogenesis and Repair Vascular Endothelial Injury in Rats Following Myocardial Infarction

**DOI:** 10.3389/fphys.2018.01940

**Published:** 2019-01-17

**Authors:** Yanjun Li, Ziwei Wang, Min Mao, Mingjing Zhao, Xiang Xiao, Weiliang Sun, Jing Guo, Chengxiang Liu, Deshuang Yang, Jiajun Qiao, Li Huang, Lin Li

**Affiliations:** ^1^Graduate School, Beijing University of Chinese Medicine, Beijing, China; ^2^China-Japan Friendship Hospital, Beijing, China; ^3^Dongzhimen Hospital, Beijing University of Chinese Medicine, Beijing, China; ^4^Department of Integrative Cardiology, China-Japan Friendship Hospital, Beijing, China; ^5^Institute of Clinical Medical Sciences, China-Japan Friendship Hospital, Beijing, China; ^6^Rizhao Hospital of Traditional Chinese Medicine, Rizhao, China

**Keywords:** velvet antler, myocardial infarction, endothelial progenitor cells, vascular endothelial injury, angiogenesis, Notch

## Abstract

**Objective:** This investigation examined the effect of velvet antler (VA) on endothelial progenitor cells (EPCs) and the associated effects to promote angiogenesis and repair vascular endothelial injury in rats with myocardial infarction (MI).

**Methods:** VA was analyzed by liquid chromatography-mass spectrometry. Male Sprague Dawley rats were randomly divided into four groups: sham, MI, VA, and VA + DAPT (gamma-secretase inhibitor IX, a specific blocker of the Notch signaling pathway) group. The rats underwent ligation of the left anterior descending coronary artery for the establishment of MI. Sham-operated rats were used as controls. Blood was taken from the orbital plexus on the first and third days after the operation, and all rats were euthanized on the 7th day after surgery. The blood samples were used to detect the contents of circulating endothelial progenitor cells (CEPCs) and vascular endothelial growth factor (VEGF). Echocardiography was used to test the cardiac function. Cardiac tissue was used for immunohistochemistry and electron microscope, and the marginal zone of the MI tissue was used for western blot and reverse transcription-quantitative polymerase chain reaction.

**Results:** The number of basically qualitative metabolites is 445. Among them, there are 74 substances with relative content greater than 0.05%. VA increased the concentration of CEPCs and VEGF in serum, CD133 content and microvessel density (MVD), and protected the morphology of microvascular endothelial cells in the marginal area of MI at 7 days post-MI surgery. CEPCs and MVD in the VA +DAPT group were lower than those of VA group. VA increased the protein expressions of Jagged-1, Notch1, NICD and HES1, and the mRNA expressions of Hes1 and Hey2, while some of the effects could be suppressed by DAPT.

**Conclusion:** These results suggest that VA promotes the mobilization of EPCs to promote angiogenesis and repair vascular endothelial cell damage in post-MI rats, and these effects may be due to activation of the Notch signal pathway.

## Introduction

Ischemic heart disease is the most common cause of death in many countries, and its frequency is increasing (Ibanez et al., 2018). The 2014 report on cardiovascular diseases in China revealed that about 2.5 million patients suffer from MI (Weiwei et al., 2016). Acute ischemia, caused by acute MI, severely damages myocardial function (Benjamin and Schneider, 2005). The key to MI treatment is to fully restore the vascular perfusion as quickly as possible. In this regard, increasing MVD and repairing endothelial injury can help restore perfusion and reduce infarct size (Galaup et al., 2012; Nie et al., 2018; Yang et al., 2018).

Endothelial progenitor cells are a type of pluripotent stem cell and usually settle in the BM ready to be mobilized to peripheral blood so that they may home to damaged sites and differentiate into mature vascular endothelial cells. This process occurs after a MI and participates in angiogenesis and promotes the repair of vascular endothelium (Asahara et al., 1999). AMI patients showed increased mobilization of EPCs from BM (Mills et al., 2009). The concentration and function of CEPCs are predictive of the prognosis of patients following AMI and are associated with the coronary collateral development and cardiovascular mortality in patients with coronary artery disease (Lambiase et al., 2004; Zeoli et al., 2009).

Early EPCs (in the BM or directly after reaching the bloodstream) are CD133+/CD34+/VEGFR2+ cells. When EPCs mobilize to peripheral blood, the expression of CD133 decreases gradually, and the expression of typical membrane molecules of mature endothelial cells, such as von Willebrand factor and platelet endothelial cell adhesion molecule-1 (PECAM-1 or CD31) increases (de la Puente et al., 2013). EPCs are rare, and the rate of their proliferation is slow, resulting in an inability to meet the need for tissue regeneration after MI. Therefore, increasing the number of EPCs has become an attractive therapeutic target for the treatment of AMI, because they can repair vascular endothelial damage and promote angiogenesis. Importantly, many signaling pathways are involved in the regulation of EPCs mobilization, homing and differentiation, and recent reports suggest that the Notch pathway is involved in inducing EPCs mobilization from BM (Kwon et al., 2009; Koide et al., 2011; Lan et al., 2017).

Velvet antler is commonly used in traditional Chinese medication and is the only mammalian organ that can fully regenerate when lost from its pedicle (Li et al., 2014). The antler can rapidly grow into a tissue rich in blood vessels, nerves, fur, and flesh within 60 days. VA contains several kinds of substances, with proteins serving as the predominant active component (Zhu et al., 2017), and previous work analyzed the proteins in VA by Nano LC-MS/MS and found that VA-protein could promote BM-derived EPCs proliferation and migration of Sprague Dawley (SD) rats *in vitro*, which might be related to the Notch and AKT/mTOR signal pathway (Xiao et al., 2017a). Previous investigation also demonstrated that 200 mg/(kg ⋅ d) of VA increases EF and FS in rats with MI-induced heart failure (Shao et al., 2012). Given these reports, we wanted to further investigate the constituents of VA, the effects of VA on the mobilization of EPCs and the after-effects in MI rats and explored the associated mechanisms. We tested the hypothesis that VA mobilizes EPCs in MI rats to promote angiogenesis and repair vascular endothelial injury via the Notch pathway. The flow of the experiment was shown in the Supplementary Figure S1.

## Materials and Methods

### VA Preparation

Velvet antler was obtained from Liaoyuan Dongfeng Sika Deer Farm, (Jilin, China). Fresh VAs were sliced, freeze-dried using a vacuum freeze dryer (-74°C and 12 Pa, LABCONCO, United States), then were comminuted by a grinder. VA powder was mixed with ultrapure water at the mass-volume ratio of 1:10, and ultrasonically extracted for 30 min. Took 50 μl supernatant, and added 450 μl precipitator (methanol: acetonitrile = 1:1). Mixed the two with turbine mixer for 60 s, then centrifuged with 13000 rpm for 10 min. Took 100 μl liquid to do LC-MS analysis.

### LC-MS Analysis

Liquid chromatography was performed and the liquid was separated with a C18 column (2.1 × 100 mm, 3 μm, Thermo Hypersil Gold) at 0.35 mL/min constant flow rate. Peptides were eluted with a gradient of 5–100% methanol and ammonium formate containded 0.1% formic acid over 30 min. The eluates were directly entered into a Q-Exactive Orbitrap Mass Spectrometer (Thermo Fisher Scientific), which was set in both positive and negative ion modes, the scan range was 75–1000 m/z and scan resolution was at 70,000. Positive ion source voltage was set at 3,500 V and negative voltage was set at 2,500 V. 2 MS/MS acquisition modes and higher collision energy dissociation were employed to evaluate the performance of this MS.

### MS Data Analysis

After LC-MS measurement, the data were collected by the Xcalibur4.1 (Thermo), and sent to OSI/SMMS (Dalian ChemDataSolution Information Technology Co. Ltd., China) (Zhao et al., 2018), the Human Metabolome Database, metlin metabolite database and the Global Natural Product Social Molecular Networking to be qualitatively analyzed. The components were scored by OSI/SMMS software and relative content of different components were compared.

**FIGURE 1:**
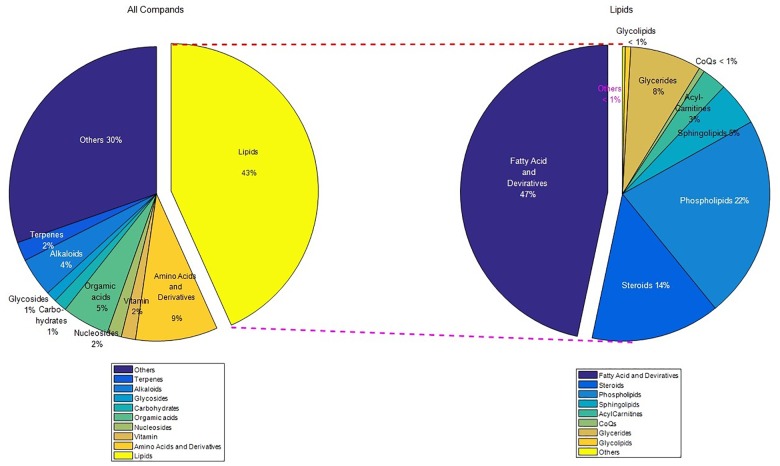
The classification map of the LC-MS result (score greater than 0.1). There are 155 kinds of fatty acids and derivatives, of which 110 are unsaturated fatty acids and derivatives. Unsaturated fatty acids and amino acids are the main components. “Others” means the classification content is less than 1%.

There are 790 components that can be basically determined the category (score greater than 0.1), of which 445 are basically qualitative (score greater than 0.5), and 279 are qualitative (score greater than 0.7) (Supplementary Table S1). Among the components with a score greater than 0.5, there are 74 substances with relative content greater than 0.05% (Supplementary Table S2), including unsaturated fatty acid, amino acids and so on. The data have been deposited to the ProteomeXchange Consortium via the iProX partner repository (Ma et al., 2018) with the dataset identifier PXD011912 (Figure 1).

### Animals

Male SD rats (weighing 230 ± 20 g) were obtained from the HuaFukang Bioscience company, Beijing, People’s Republic of China. All procedures complied with the Animal Management Rule of the Ministry of Public Health, People’s Republic of China (Document No. 55, 2001). All experimental protocols were reviewed and approved by the Animal Care & Welfare Committee Review Comment of China-Japanese Friendship Hospital (Permit Number: 17-0106).

### Establishment of MI Model and Group

A total of 38 rats, weighing 230–250 g, were randomly divided into four groups: sham group (*n* = 8), model group (*n* = 10), VA (*n* = 10), VA +DAPT group (*n* = 10). In the sham and model groups, the rats were given 0.5% sodium salt of carboxymethyl cellulose (CMC-Na) (Solarbio, China) once a day. In the VA group, the rats were given 400 mg/(kg ⋅ d) VA (dissolved in 0.5% CMC-Na) by gavage. In the VA +DAPT group, the rats were given 400 mg/(kg ⋅ d) VA and 10 mg/(kg ⋅ d) DAPT (Selleck, China, dissolved in 4% DMSO and corn oil) via gavage 30 min before experimentation and then once per day until the end of the experiment (Yin et al., 2015). DMSO (Solarbio, China) and corn oil (Sigma, United States), the vehicle for DAPT, were administered in the same manner in the sham, model and VA groups.

The rats were anesthetized with 1% pentobarbital (50 mg/kg) given by intraperitoneal injection before surgery. Rats were then endotracheally intubated and mechanically ventilated. An incision was then made along the left sternal border, and a horizontal incision was made between the fourth and fifth rib to open the chest and expose the heart. Then, the left anterior descending branch of coronary artery was ligated 1.5–2 mm from the left atrium margin between the pulmonary cone and the left auricular appendage with a 2-0 suture. Upon ligation, the anterior wall and the apex of the heart below the ligation point became white and demonstrated significant hypomotility. Then, the muscles and skin were sutured closed, and the mechanical ventilation was terminated when rats’ respiration and heart rate became stable. The sham group experienced the identical procedures except for coronary artery ligation. After the surgery, all rats were injected with 400,000 U penicillin to prevent infection. After modeling, an electrocardiogram was given to test the infarction degree. Pathologic Q-waves from I, avL, V3–V6 and V1 or V2, indicated that the establishment of the MI model was successful (Wang et al., 2017).

After the surgery, five rats died of heart failure or arrhythmia, and one was excluded due to infarct area limitation. The remaining rats in each group: sham (*n* = 8), model (*n* = 8), VA (*n* = 8), and VA +DAPT (*n* = 8). Blood was taken from the inner canthus at the first and third days after the operation, and all rats were euthanized on the 7th day after the surgery.

### Measurement of Cardiac Structural and Functional Parameters

Echocardiography (Vivid E9, GE, United States) was performed before the rats were euthanized. For this measurement, the rats were anesthetized with a intraperitoneal injection of sodium pentobarbital, fixed on the operating table in the supine position, and the hair of the chest was shaved. According to the American Society of Echocardiology leading-edge method (Lang et al., 2015), EF and FS were calculated. Three consecutive cardiac cycles were measured in each rat.

### Evaluation of CEPCs

Rats were anesthetized by intraperitoneal injection of 1% pentobarbital sodium (50 mg/kg). At the first and third days after the operation, blood was collected from the inner canthus and into EDTA coated tubes for anticoagulation. On the 7th day after operation, all the rats were sacrificed, and blood was collected from the abdominal aorta in EDTA vacuum collecting tubes. Five hundred microliters of blood and 3.5 ml red cell lysate were mixed to prepare a cell suspension. Marrow-derived EPCs in circulating blood were characterized by assessing the surface markers. PE-conjugated anti-CD34 (Bioss, China), PE-CY7 conjugated anti-CD133 (Bioss), and FITC conjugated anti- VEGFR2 antibody (Bioss) were used. CEPCs were analyzed by quantitative 3-color flow cytometry using a fluorescence-activated cell sorter (FACSCanto TM II, BD, United States). The data were collected from 100,000 cells per sample.

### Enzyme-Linked Immunosorbent Assay (ELISA)

Before the rats were sacrificed,5ml of blood was collected from the abdominal aorta and centrifuged at 1,800 ×*g* for 15 min. The supernatant was collected and stored at -20°C. The contents of VEGF were measured by an ELISA kit (Bioss, China) according to the manufacturer’s instructions.

### Immunofluorescence

The heart was removed, rinsed with normal saline, sliced into 4 μm sections, and frozen immediately. The sections were washed three times with PBS and blocked with goat serum for 30 min at room temperature. Then the sections were incubated with rabbit polyclonal antibody to CD133 (ab19898, Abcam, United Kingdom) and mouse monoclonal antibody to CD31 (ab64543, Abcam, United Kingdom) at 4°C overnight. The next day sections were incubated with fluorescent-labeled secondary antibodies: anti-rabbit IgG (Alexa Fluor 488 Conjugate, ab150077, Abcam, United Kingdom), and anti-mouse IgG (Alexa Fluor 555 Conjugate, #4409, CST, United States) in the dark for 60 min at room temperature. Finally, the sections were washed and sealed with an anti-fluorescence quench agent which contained with 4′,6-Diamidino-2-phenylindole (ab104139, Abcam, United Kingdom). Images were acquired with a fluorescence microscope (Olympus, Japan) using a ×400 magnification. Color composite images were generated with Image Pro-plus software.

### Electron Microscopy

The border zone of infarcted myocardium was fixed with 2.5% glutaraldehyde and after-fixed with 1% osmic acid. These sections were then dehydrated with acetone, and embedded in epoxy resin 812, and made into ultra-thin sections before being stained with uranyl acetate and lead citrate. The ultrastructure of the myocardium was then observed by transmission electron microscopy (JEM-1400 plus; JEOL., Tokyo, Japan).

### Western Blot

Protein was extracted from the myocardium around the infarcted area (Solarbio, China), and the concentration was measured using a bicinchoninic acid protein concentration kit (Beyotime, China). The protein samples were separated by 10% acrylamide gel electrophoresis and transferred to nitrocellulose membranes, which were later incubated in the first antibody at 4°C overnight. The following target protein was detected to obtain their expression level: Jagged-1 (ab109536, Abcam, United Kingdom), Notch1 (3608, CST, United States), NICD (ab52301, Abcam, United Kingdom), Hes-1 (11988, CST, United States), GAPDH (Hangzhou Good Here Biotechnology, China). After the membranes were washed three times with PBS-T (PBS containing 0.5% Tween 20), they were further incubated in the secondary antibody (Jackson ImmunoResearch, United States) for 1 h at room temperature away from light. Then, bands were acquired using ECL visualization. The images were analyzed by the Image-J software.

### RT-qPCR

Total RNA was extracted from the ischemic border zone of rat cardiac using Easyspin Plus RNA Extraction Kit (Aidlab, Beijing, China) according to the manufacturer’s protocol. Total RNA was reverse-transcribed into cDNAs and amplified using a iScript^TM^ cDNA Synthesis Kit (Bio-Rad Co., Ltd., United States). A real-time PCR kit (Taq SYBR^®^ Green qPCR Premix, PROMEGA) was used to measure expression levels. PCR efficiency was examined, and the melting curve data was collected for PCR specificity. Relative mRNA levels were determined to those of GAPDH and are analyzed with the 2ˆ(-Delta Delta CT) method. Statistical analysis was done with a Mann–Whitney *U* test at a 95% confidence interval. The primers used for real-time PCR were as follows: forward, 5′-TGG AGA GGC TGC CAA GGT T-3′ and reverse, 5′-AGG CGA CAC TGC GTT AGG AC-3′ for Hes1; forward, 5′-GTC AAC GCC ATG TCG CCT AT-3′ and reverse, 5′-TGA TGG CAT CCG AAG AGC AG-3′ for Hey2; forward, 5′-CAT TCT TCC ACC TTT GAT-3′ and reverse, 5′-CTG TAG CCA TAT TCA TTG T-3′ for GAPDH.

### Statistical Analysis

All data are expressed as mean ± the standard deviation (SD). Comparisons between groups were performed by one-way analysis of variance (ANOVA) with the least significant difference (LSD) test where appropriate. Statistical significance was defined as *P <* 0.05. Analyses were carried out using SPSS 17.0 Software.

## Results

### Changes of Electrocardiogram and Echocardiography in Rats

**FIGURE 2:**
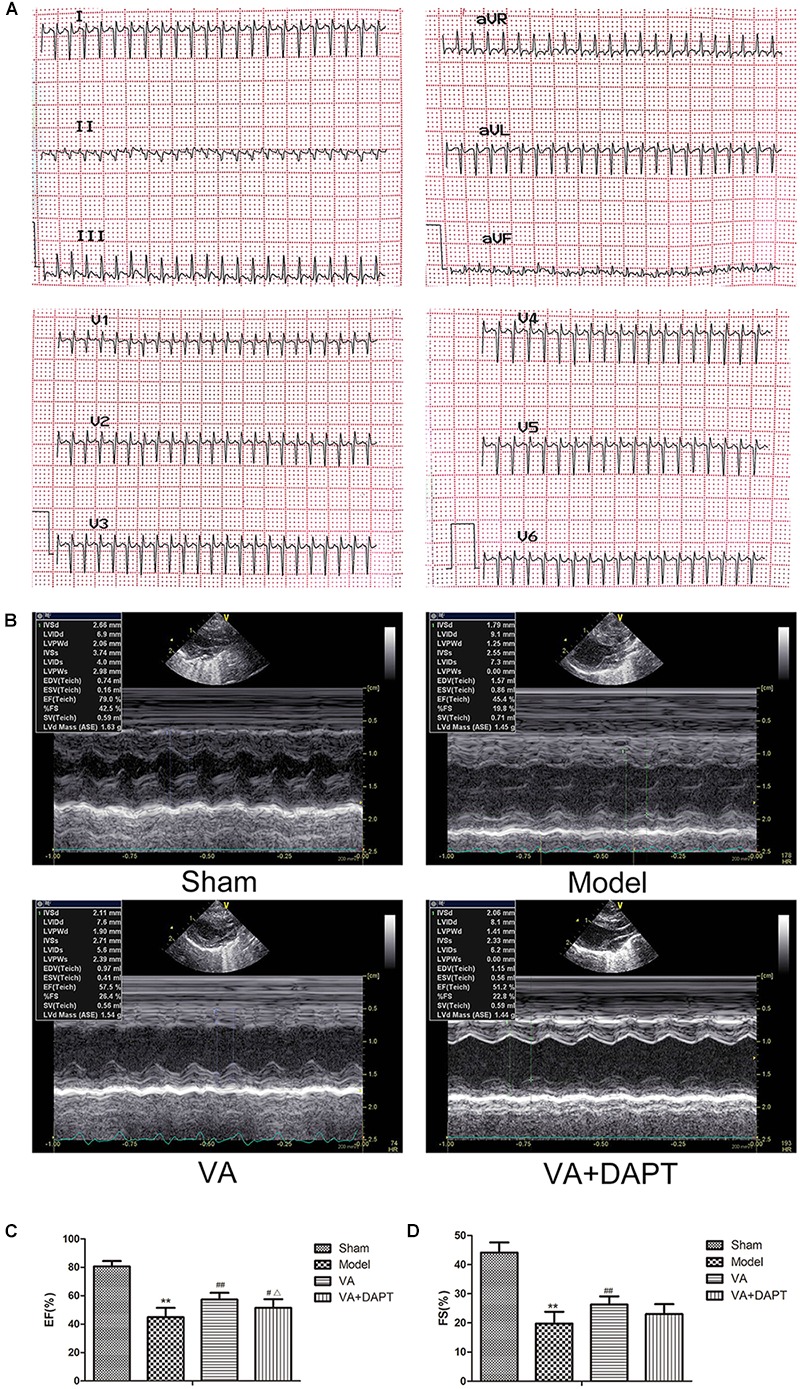
Changes of electrocardiogram and echocardiography in rats. **(A)** Q waves indicated that the surgery was successful. **(B)** Left ventricular long-axis views 7 days after MI. **(C)** Left ventricular ejection fraction (EF). **(D)** Left ventricular fractional shortening (FS). MI resulted in a visible drop in the ultrasonic changes in the model group, and the ultrasonic changes in VA groups were alleviated compared with the model group. ^∗∗^*P* < 0.01 compared with the sham group; ^#^*P* < 0.05 and ^##^*P* < 0.01 compared with the model group; ^Δ^ *P* < 0.05 compared with the VA group. *N* = 8.

After the surgery, Q-waves from I, avL, V3–V6 and V1 or V2 indicated that the surgery was successful. One rat was eliminated because it did not meet the standard for the electrocardiogram (Figure 2A). Seven days after the surgery, there were some differences in the left ventricular long-axis views (Figures 2B). MI caused a significant decrease in EF and FS in the model group compared with the sham group (*P <* 0.01). VA significantly increased EF and FS compared with the model group (*P <* 0.01), and the effects were suppressed by DAPT (Figures 2C,D).

### Detection of CEPCs Content by Flow Cytometry

**FIGURE 3:**
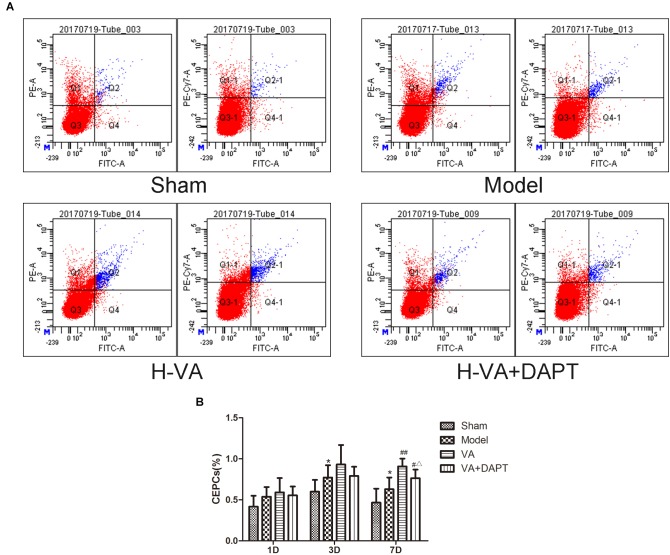
CEPCs at different times after MI. **(A)** Flowcharts of each group on day 7 after MI. **(B)** CEPCs at different time after MI. ^∗^*P* < 0.01 compared with the sham group; ^#^*P* < 0.05 and ^##^*P* < 0.01 compared with the model group; ^Δ^ *P* < 0.05 compared with the VA group. *N* = 8.

CEPCs were marked by CD34, VEGFR2, and CD133 (Figure 3A). Three days after MI, the concentration of CEPCs in model group was higher than that in the sham group (*P <* 0.05). Seven days after MI, CEPCs in model group was still higher than that in the sham group (*P <* 0.05), and the concentration of CEPCs in the VA group was greater than the model group (*P <* 0.01). CEPCs in the VA +DAPT group was lower than that of VA group (*P <* 0.05), and higher than that of the model group (*P <* 0.05) (Figure 3B).

### Levels of VEGF in Serum

**FIGURE 4:**
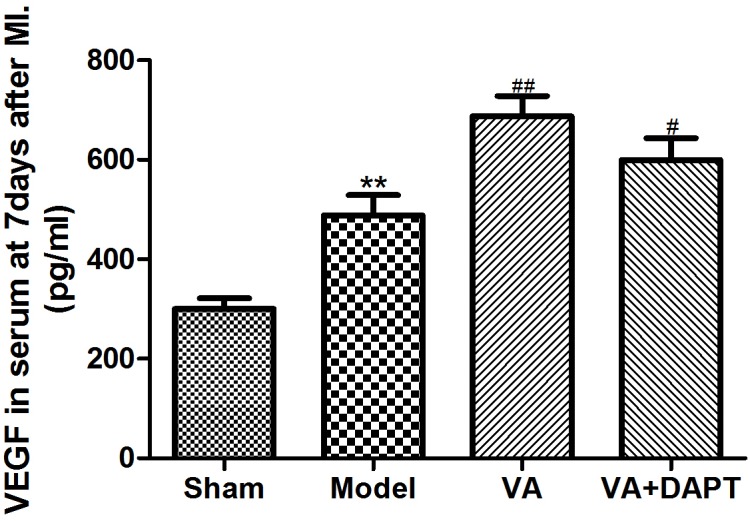
Levels of VEGF in serum at 7 days after MI. ^∗∗^*P* < 0.01 compared with the sham group; ^#^*P* < 0.05 and ^##^*P* < 0.01compared with the model group.

Seven days after the surgery, the level of VEGF in serum increased in model group compared to sham group (*P* < 0.01), and the level of VEGF raised in VA group than that in model group (*P* < 0.01). VEGF in the VA +DAPT group was also higher than that in the model group (*P <* 0.01) (Figure 4).

### CD133 and MVD in the Border Zone of MI by Immunofluorescence

**FIGURE 5:**
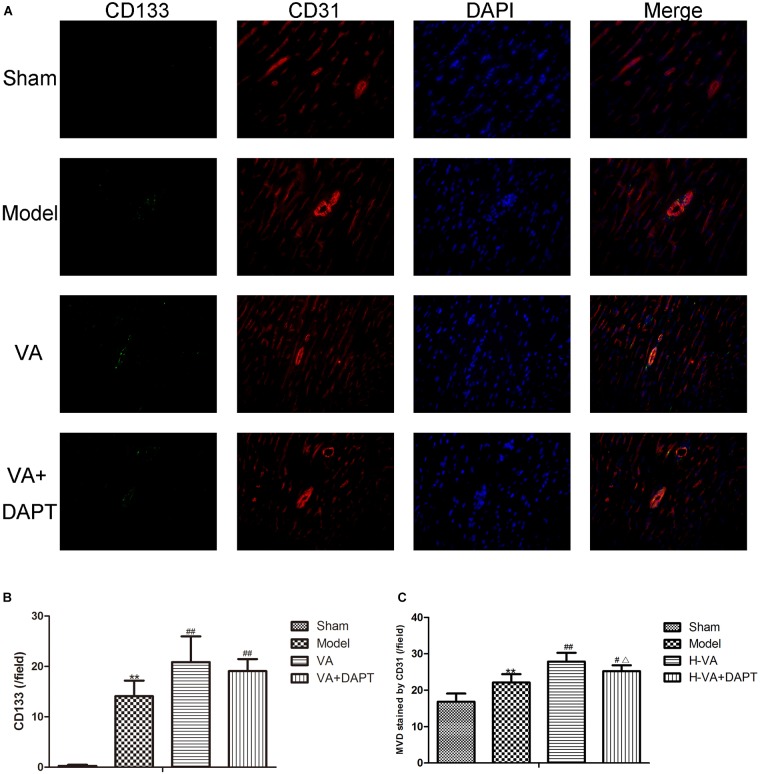
CD133 and MVD in the border zone of MI. **(A)** Immunofluorescence on days 7 after MI. **(B)** Expression of CD133 stained by immunofluorescence (per/field). **(C)** MVD stained by CD31 (per/field). CD133 present star like and distribute on vessels marked by CD31, suggesting that angiogenesis might be promoted by EPCs marked by CD133. ^∗∗^*P* < 0.01 compared with the sham group; ^#^*P* < 0.05 and ^##^*P* < 0.01 compared with the model group; ^Δ^ *P* < 0.05 compared with the VA group. *N* = 8.

Seven days after the surgery, the CD133 content in the marginal zone of the MI group was higher than that of the sham group (*P <* 0.01), and the CD133 of VA group was higher than that of the MI group (*P <* 0.01). CD133 in the VA +DAPT group was also higher than that in the MI group (*P <* 0.01), but there was no difference of CD133 in the VA and the VA +DAPT group (Figure 5).

Seven days after the surgery, the MVD stained by CD31 in the MI group was higher than that of the sham group (*P <* 0.01), and the MVD stained by CD31 in the VA group was higher than that of the MI group (*P <* 0.01). The MVD stained by CD31 in the VA +DAPT group was less than the VA group (*P <* 0.05), and higher than that of the MI group (*P <* 0.05, Figure 5).

### Electron Microscope Pictures of Vascular Endothelial Cells in the Marginal Area of MI

**FIGURE 6:**
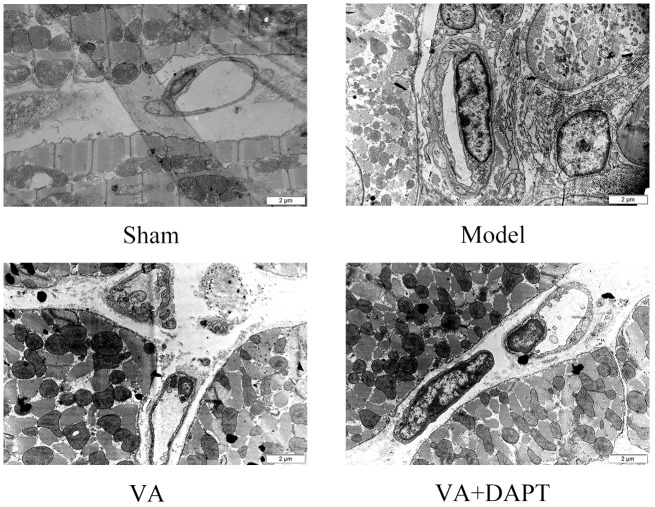
The morphology of microvascular endothelial cells in the marginal area of MI 12000×.

Seven days after surgery, the endothelial cells in the sham group were normal, with no obvious swelling and no thickening of the basement membrane. However, in the MI group, the endothelial cell nuclei were swollen and convex to the lumen, resulting in narrowing of the lumen and thickening of the basement membrane. Compared with the model group, the pathological changes of vascular endothelial cells in the VA group were reduced, the swelling was lighter, the lumen was larger, and the basement membrane thickening was not as evident (Figure 6). More pictures of different rats can be seen in the Supplementary Figures S2, S3.

### The Protein Expressions of Jagged-1, Notch1, NICD, and HES1 in the Marginal Zone of MI

**FIGURE 7:**
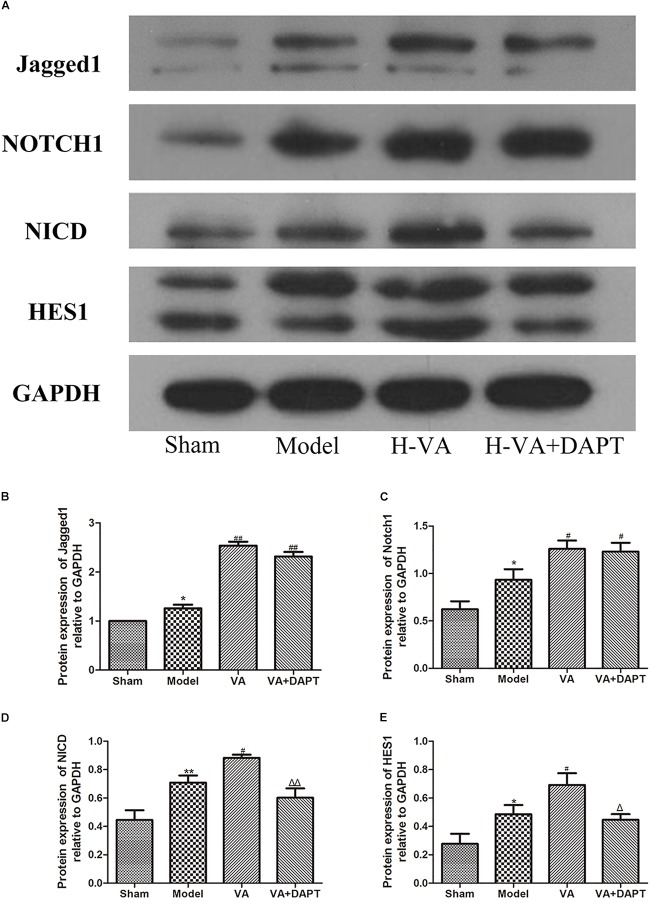
Expressions of Notch pathway related proteins in the border zone of MI. **(A)** Bands of different proteins. **(B–E)** Protein expressions of Jagged-1, Notch1, NICD and HES1 relative to GAPDH, respectively. ^∗^*P* < 0.05 and ^∗∗^*P* < 0.01 compared with the sham group; ^#^*P* < 0.05 and ^##^*P* < 0.01 compared with the model group; ^Δ^ *P* < 0.05 and ^ΔΔ^ *P* < 0.01 compared with the VA group. *N* = 5.

Seven days after surgery, the protein expression of Jagged-1 in the marginal area in the VA and VA +DAPT group was higher than that of the MI group (*P <* 0.01). The protein expression of Notch1 in the marginal zone of the MI group was higher than that of the sham operation group (*P <* 0.01). The Notch1 in the VA and VA +DAPT group was higher than that of the model group (*P <* 0.05). The protein expression of NICD protein in the marginal zone of the MI group was higher than that in the sham group (*P <* 0.01), and the NICD in VA group was higher than that in the model group (*P <* 0.05). The NICD in the VA +DAPT group was less than the VA group (*P <* 0.01). The trend of HES1 was similar to that of NICD (Figure 7). More protein bands of different rats can be seen in the Supplementary Figures S4, S5.

### The mRNA Expressions of Hes1 and Hey2 in the Marginal Zone of MI

**FIGURE 8:**
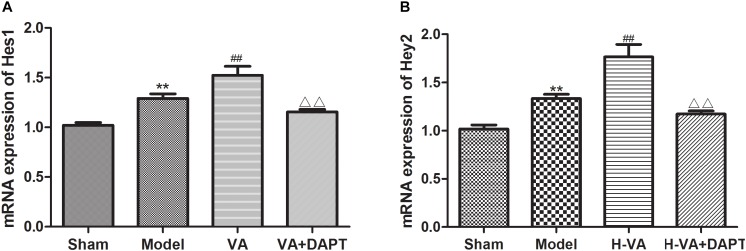
mRNA expression levels of Hes1 and Hey2 in the border zone of MI. **(A,B)** mRNA expressions of Hes1 and Hey2 relative to GAPDH, respectively. ^∗∗^*P* < 0.01 compared with the sham group; ^##^*P* < 0.01 compared with the model group; ^ΔΔ^ *P* < 0.01 compared with the VA group. *N* = 8.

Seven days after surgery, the mRNA expression of Hes1 in the marginal zone of MI in the MI group was more than that of the sham group (*P <* 0.01). The mRNA expression of Hes1 in the VA group was higher than that in the MI group (*P <* 0.01), and the effect of VA could be suppressed by DAPT (*P <* 0.01). The trend of Hey2 was the same with Hes1 (Figure 8).

## Discussion

### VA Regulated EPCs in MI Rats to Promote Angiogenesis and Vascular Endothelial Repair

Velvet antler contains several kinds of ingredients. Previous work analyzed the macromolecular proteins in VA by Nano LC-MS/MS and found that VA-protein, the main component of VA, promotes BM- derived EPCs proliferation and migration of SD rats *in vitro* (Xiao et al., 2017a). LC-MS can examine the small molecules like amino acids and fatty acids in VA. Among these detected amino acids, arginine pretreatment could enhance CEPCs population in high-fat diet-induced obese mice with limb ischemia (Kuo et al., 2018), and promotes EPCs mobilization in mice with polymicrobial sepsis (Yeh et al., 2017). Glutamine supplementation could enhance EPCs mobilization in streptozotocin-induced diabetic mice subjected to limb ischemia (Su et al., 2017). Replacement of dietary saturated fat with unsaturated fats increases numbers of CEPCs in United Kingdom adults with moderate cardiovascular disease risk (Weech et al., 2018).

The effectiveness of VA has been confirmed though complex composition. Changes in the echocardiography support the notion that VA improves cardiac function. Flow cytometry in this experiment indicated that VA significantly increased BM-derived EPCs in circulating blood in rats after MI, which may be related to mobilization of EPCs in BM. In addition, some researchers found that VA could mobilize EPCs in lower limb ischemia rats, promote angiogenesis and reduce the ischemia of the affected limbs, which is consistent with the results reported herein (Yi-Ran and Ting-Quan, 2017). While further researches are still required to investigate VA.

CD133, a surface marker of EPCs, is gradually degraded after entering the peripheral blood. Compared with the MI group, the increase of CD133 content in the edge region of MI, maybe a follow-up to the increase of CEPCs, or because VA protein could promote the migration of EPCs (Xiao et al., 2017a).

Velvet antler increased the MVD stained by CD31 in the edge region of MI. CD31 is expressed on the cell surface of endothelial cells and increased expression of CD31 indicates an effective increase in MVD and a higher level of angiogenesis (Weil et al., 2011). CD133 present star like and distribute on vessels marked by CD31, suggesting that angiogenesis might be promoted by EPCs marked by CD133. VA also increased the content of VEGF in serum, which can stimulate angiogenesis (Pohl-Schickinger et al., 2010). The morphology of vascular endothelial cells in the VA treated group was better than that in the MI group, which is consistent with previous work, that is, VA proteins had protective effects on the cardiac microvascular endothelial cells challenged with ischemia-hypoxia (Xiao et al., 2017b). These results suggest that VA could promote angiogenesis and repair the damaged vascular endothelium in rats with MI by regulating EPCs, and these may save ischemic myocardium and result in an improvement of cardiac function. However, experiments are needed to explore the mechanism of action.

### Mechanism of VA to Regulate EPCs

The Notch signaling pathway can affect and regulate the mobilization, *in situ* differentiation and integration of EPCs with the target tissues. There are four membrane protein receptors (Notch1-4) and five ligands (Jagged1-2 and Delta-like 1, -3,-4) in mammals. The interaction of receptors with the ligands, leads to the hydrolysis of the transmembrane Notch receptor under the action of γ-secretase, producing the Notch intracellular segment (NICD). NICD directly enters the nucleus and regulates the expression of the target gene, the hairy and enhancer of split (Hes) and has-related (Hey), and then regulates the biological activity of the cells (Rizzo et al., 2013).

Jagged1-derived Notch signaling could affect the microenvironment of BM, and affect the proliferation, migration, mobilization, and differentiation of BM EPCs, which is of vital importance to the angiogenesis mediated by EPCs (Kwon et al., 2008). Loss of the expression and function of Jagged1 ligands leads to (a) the decrease in the EPC colony of BM; (b) the decline of proliferation, migration and activity of EPCs from BM and (c) the dysfunction of EPCs in the treatment of vascular and ischemic tissue regeneration (Kwon et al., 2009). Notch1 positive EPCs was more active than wild-type EPCs, and Notch1 positive EPCs could accelerate recovery after endothelial injury in wild-type mice (Ii et al., 2010).

Seven days after MI, CEPCs and the protein expression of Jagged-1, Notch1, NICD, Hes1, mRNA expression of Hes1 and Hey2 in the marginal zone of MI in the VA treated group was higher than those in the MI group, suggesting that VA may promote the mobilization of EPCs through the Notch pathway. The content of CEPCs and MVD in the VA +DAPT group were lower than that in the VA group. DAPT, a gamma-secretase inhibitor, which does not affect ligand Jagged1 and receptor Notch1, could inhibit the expression of NICD and subsequent reactions. The concentration of Jagged1 and Notch1 in the marginal area of MI in the VA+DAPT group was not different from that in the VA group, but NICD, Hes1, and Hey2 were less than those in the VA group. These also suggested that VA might promote the mobilization of EPCs through the Notch pathway. However, CEPCs in the VA +DAPT group were still higher than the model group, and we speculated VA may promote mobilization through VEGF or other signaling pathways, as VEGF also participate in the mobilization of EPCs after MI (Lan et al., 2017).

## Conclusion

Velvet antler could promote the mobilization of EPCs to promote angiogenesis and repair vascular endothelial cell damage in rats after MI, which might be related to Notch pathways.

## Author Contributions

YL and ZW carried out the experiments and wrote the manuscript. MM, XX, LL, and LH designed the experiments and revised the primary manuscript. MZ supervised the study. WS and JG assisted in the completion of pathological experiments. CL wrote part of the manuscript and edited the figures. DY and JQ participated in the animal experiments.

## Conflict of Interest Statement

The authors declare that the research was conducted in the absence of any commercial or financial relationships that could be construed as a potential conflict of interest.

## Supplementary Material

Figure S1 Flow chart of the experiment.

Figure S2 The morphology of microvascular endothelial cells in the marginal area of MI.

Figure S3 The morphology of microvascular endothelial cells in the marginal area of MI.

Figure S4 Protein bands of different rats.

Figure S5 Protein bands of different rats.

TABLE S1 LC-MS analysis (score greater than 0.1).

TABLE S2 LC-MS analysis (relative content greater than 0.05%).

## Abbreviations

AMI: acute myocardial infarction
BM: bone marrow
CD31: platelet endothelial cell adhesion molecule-1
CEPCs: circulating endothelial progenitor cells
EF: left ventricular ejection fraction
EPCs: endothelial progenitor cells
FS: left ventricular fractional shortening
LC-MS: liquid chromatography-mass spectrometry
MI: myocardial infarction
MVD: microvessel density
NICD: Notch intra Cellular domain
VA: velvet antler
VEGF: vascular endothelial growth factor
