# Supplementary figures and images for: Velvet Antler Mobilizes Endothelial Progenitor Cells to Promote Angiogenesis and Repair Vascular Endothelial Injury in Rats Following Myocardial Infarction
Source: Front Physiol. 2019 Jan 17;9:1940. doi: 10.3389/fphys.2018.01940 (PMC6344410; doi:10.3389/fphys.2018.01940)

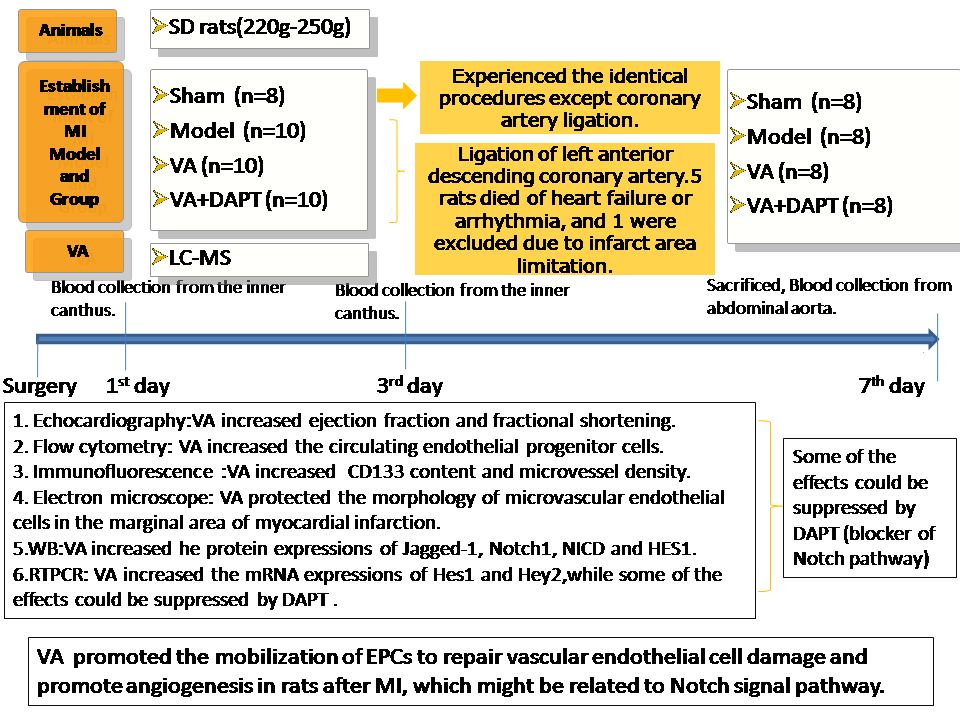

Supplement: Figure S1 — Flow chart of the experiment. [file Image_1.tif]

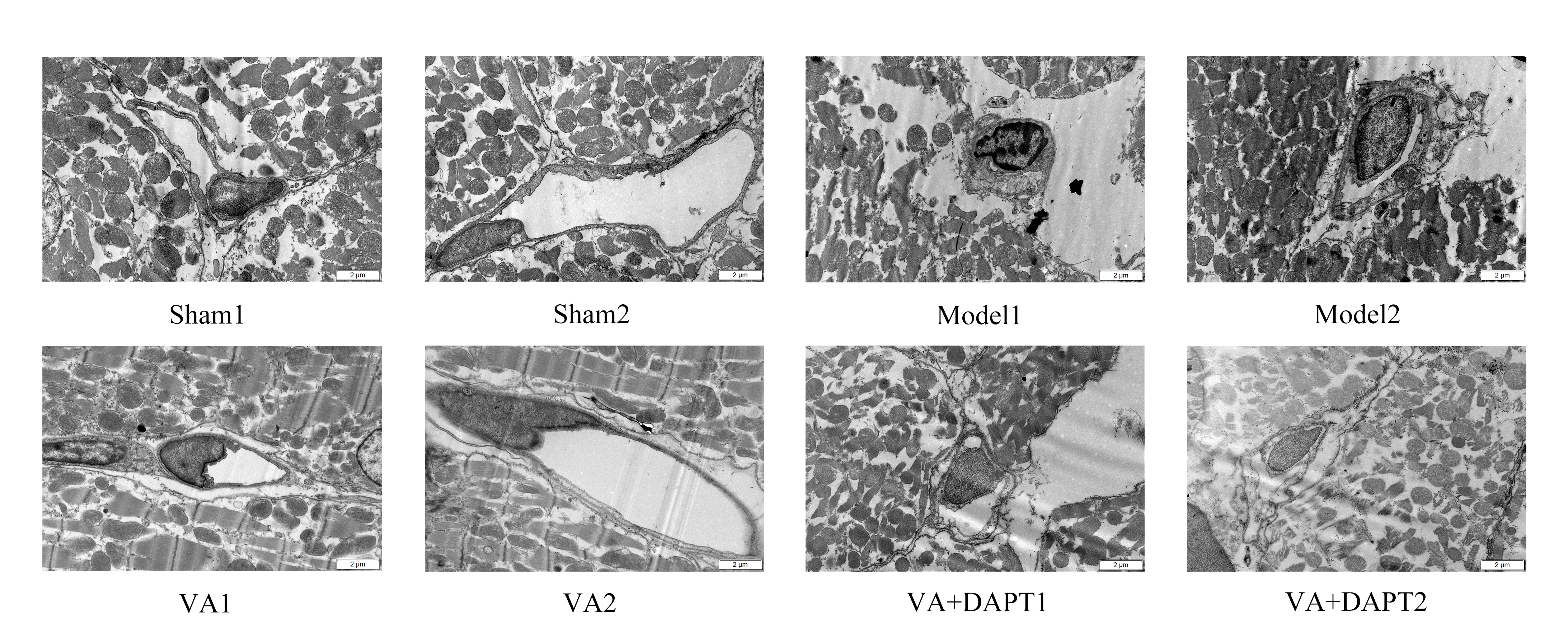

Supplement: Figure S2 — The morphology of microvascular endothelial cells in the marginal area of MI. [file Image_2.jpeg]

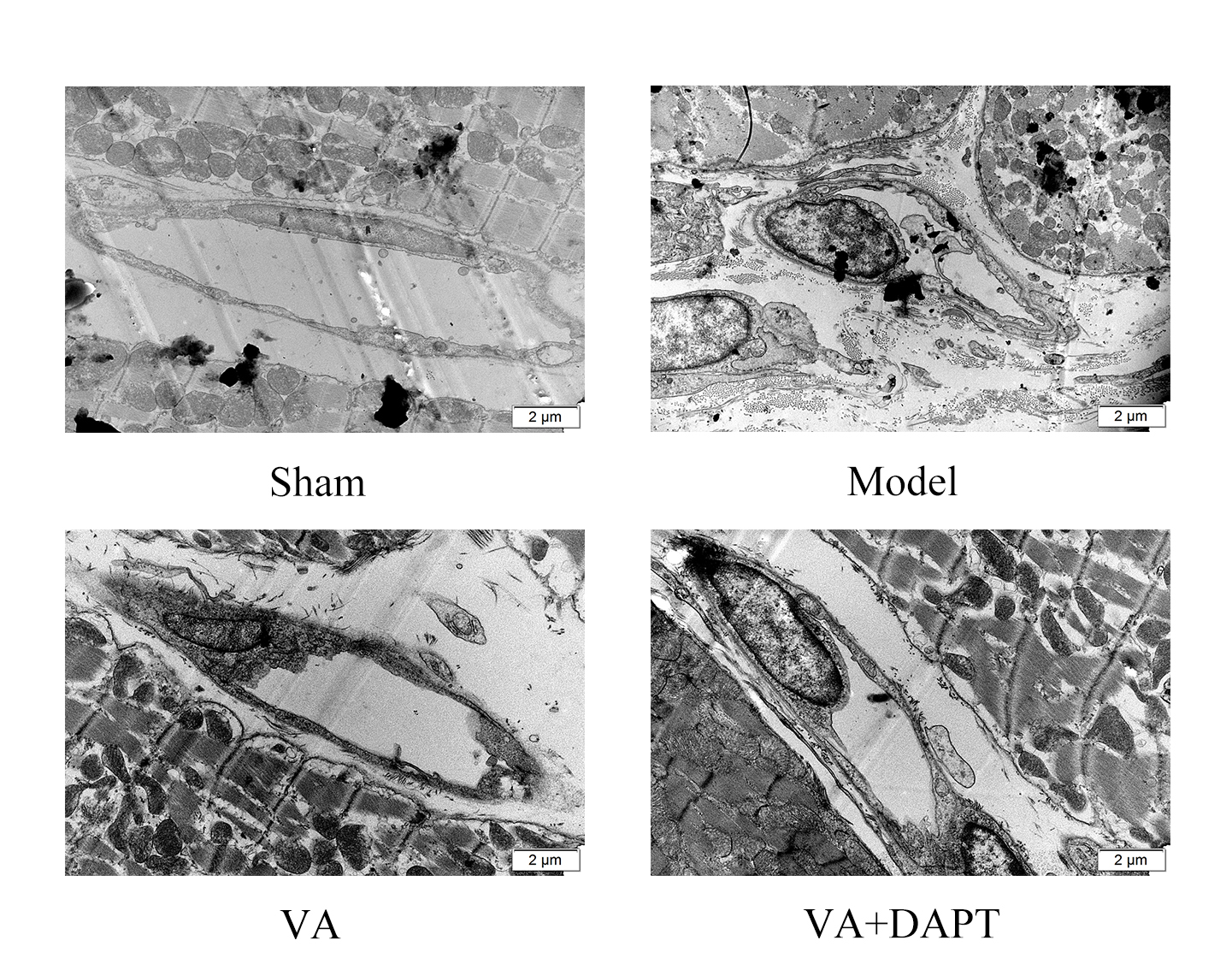

Supplement: Figure S3 — The morphology of microvascular endothelial cells in the marginal area of MI. [file Image_3.jpeg]

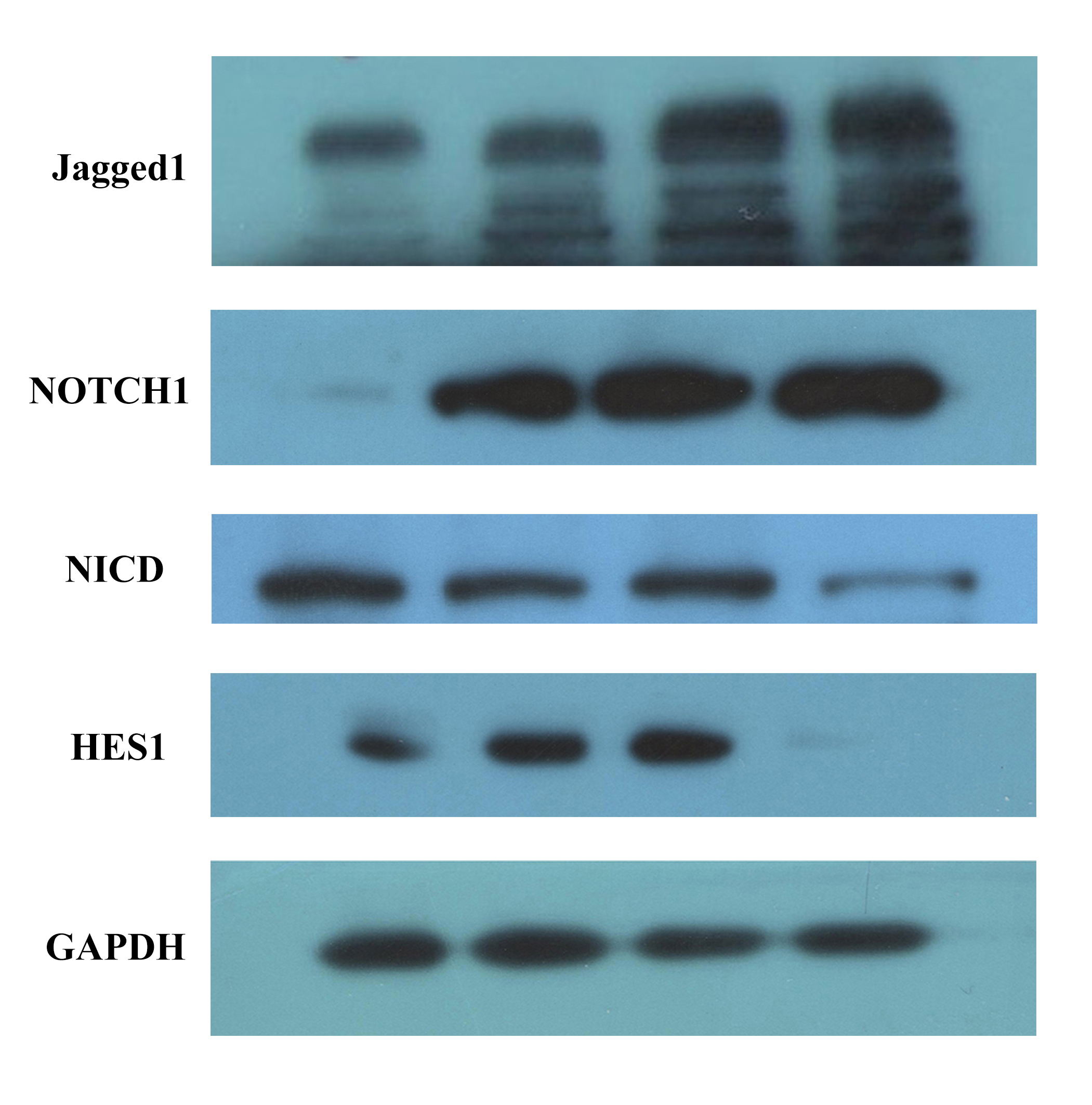

Supplement: Figure S4 — Protein bands of different rats. [file Image_4.jpeg]

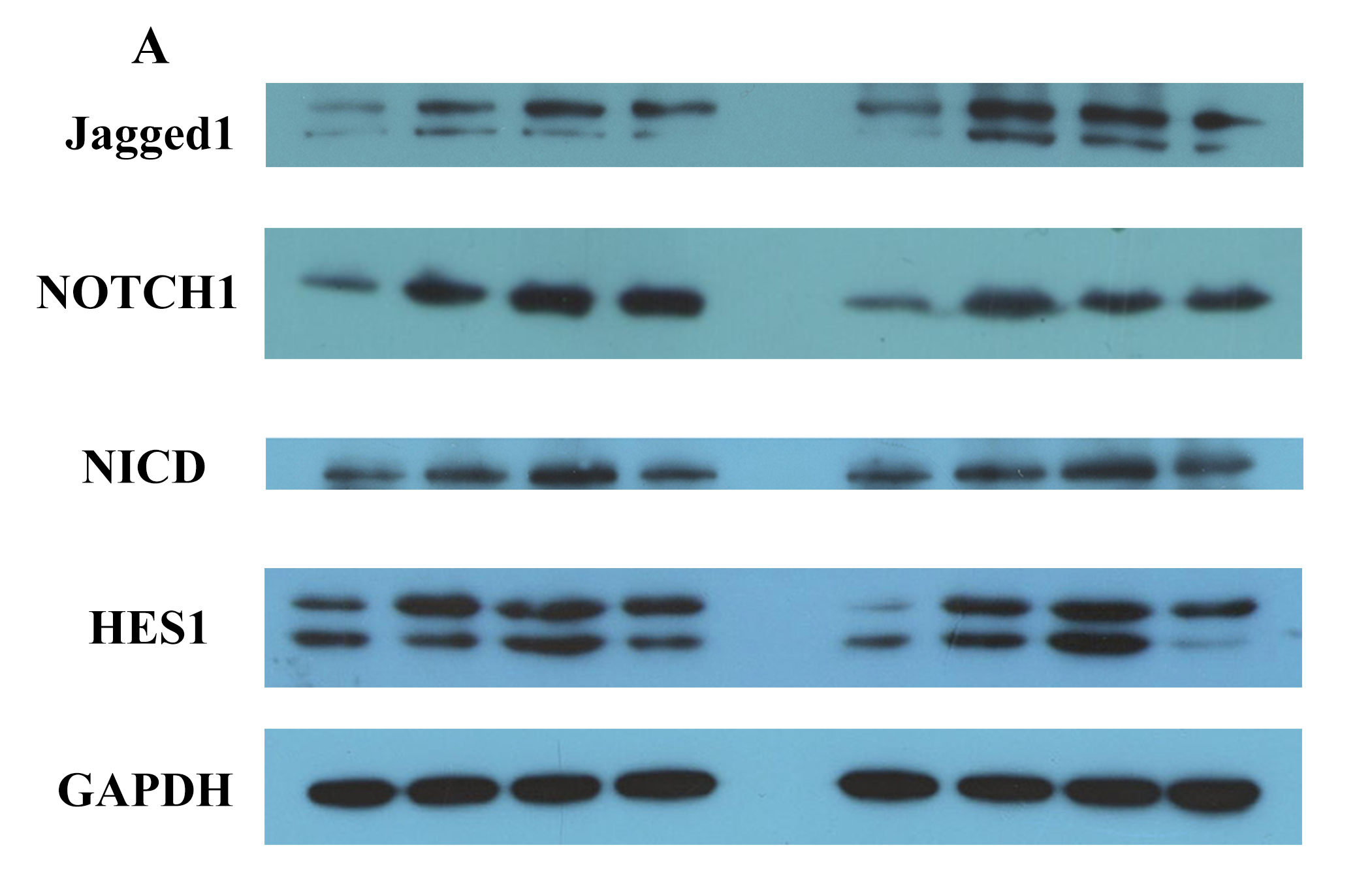

Supplement: Figure S5 — Protein bands of different rats. [file Image_5.jpeg]
